# Subtalar Joint Pronation and Energy Absorption Requirements During Walking are Related to Tibialis Posterior Tendinous Tissue Strain

**DOI:** 10.1038/s41598-017-17771-7

**Published:** 2017-12-20

**Authors:** Jayishni N. Maharaj, Andrew G. Cresswell, Glen A. Lichtwark

**Affiliations:** The University of Queensland, School of Human Movement and Nutrition Sciences, Centre for Sensorimotor Neuroscience, Brisbane, 4072 Queensland Australia

## Abstract

During human walking, the tibialis posterior (TP) tendon absorbs energy in early stance as the subtalar joint (STJ) pronates. However, it remains unclear whether an increase in energy absorption between individuals, possibly a result of larger STJ pronation displacement, is fulfilled by greater magnitudes of TP tendon or muscle fascicle strain. By collecting direct measurements of muscle fascicle length (ultrasound), MTU length (3D motion capture and musculoskeletal modelling), and TP muscle activation (intramuscular electromyography) we endeavoured to illustrate that the TP tendinous tissue fulfils the requirements for energy absorption at the STJ as a result of an increase in muscle force production. While a significant relationship between TP tendon strain, energy absorption at the STJ (R^2^ = 0.53, P = < 0.01) and STJ pronation (R^2^ = 0.53, P = < 0.01) was evident, we failed to find any significant associations between tendon strain and surrogate measure of TP muscle force (TP muscle activation together with ankle and subtalar joint moments). These results suggest that TP tendon compliance may explain the variance in pronation and energy absorption at the STJ. Therefore, as the tendinous tissue of the TP is accountable for the absorption of energy at the STJ it may be predisposed to strain-induced injury.

## Introduction

The human foot is a flexible structure designed to store and release elastic strain energy during gait^1^. As the foot collides with the ground, there is a requirement for the limb to absorb mechanical energy. Energy absorption at the foot is performed by compression of the longitudinal arch^1,2^ and active resistance to subtalar joint (STJ) pronation^3^. Pronation of the rear-foot is highly variable across the population^4^ and is linked to the amount of energy (negative work) absorbed at the STJ. However, little attention has been paid to the variance in energy absorption at the STJ between individuals, possibly a result of STJ pronation displacement, and the mechanisms and structures involved. It remains unclear whether greater energy absorption at the STJ between individuals is due to greater forces required to resist larger STJ pronation displacement or altered tissue properties (i.e. compliance) enabling greater pronation displacement and energy absorption at the STJ.

Energy absorption at the foot may be dissipative or elastic in nature. Viscoelastic tissues, such as tendons, aponeuroses, ligaments and other connective tissues can store and return energy depending on their material make-up. In particular, the elastic properties of the long tendons in the distal lower limb of humans have been highlighted as structures that likely recycle significant amounts of energy in an elastic manner during locomotion. Tendons stretch in proportion to the force applied and store elastic energy that can be returned when the load is removed, losing a small proportion of this energy (~7–10%) as heat, primarily due to its viscous properties. While no additional work is achievable through this mechanism, stretch and recoil of tendons allows the associated muscle fibres to operate at favourable lengths and velocities for force generation^5,6^, such that it can enhance the system’s economy in supporting body weight and generating the required propulsion^7^.

Our current understanding of the role of human muscles and tendons in absorbing energy or generating work is typically gleaned from muscle tendon units (MTU) that primarily operate in the sagittal plane^1,2,5,8,9^. However, energy can also be absorbed and returned in the frontal plane, particularly at the STJ. We have recently demonstrated that the primary driver of STJ supination, the tibialis posterior (TP) muscle^10^, is sufficiently compliant to absorb and return considerable energy during the stance phase of walking^3^. In early stance, there is a requirement for absorption of energy by the TP MTU, which is achieved through an increase in the STJ supination moment (via increased TP muscle activation) to resist STJ pronation. During this period, the TP tendon is stretched and is responsible for the majority of the energy that is absorbed, while the fascicles are able to activate and achieve a relatively isometric contraction. Isometric behaviour during force development is beneficial as it may allow the TP muscle to generate high forces based on the force-velocity relationship, avoid muscle injury from repetitive lengthening, and potentially improve the energetics of the contraction by reducing the volume of activated muscle to generate force^3,11^. However, it remains unclear whether increases in absorption of energy at the STJ across the population are solely absorbed within the tendinous tissues of the TP or whether the muscle fibres also absorb energy.

During movement, energy can be absorbed by either the muscle (fascicles) or the tendinous tissues. Increased elastic energy storage in the TP tendon may be the result of greater muscle force generation, which in turn may be due to differences in TP activation parameters (e.g. the timing and volume of muscle fibres recruited). Alternatively, greater TP tendon stretch could also be a function of the mechanical properties of its elastic tissues. For example, if the compliance of two TP tendons are disparate and similar forces are applied, the more compliant tendon could absorb more energy, allowing greater pronation compared to the less compliant (stiffer) tendon. In contrast, dissipation of energy by the contractile tissue could occur if there is a reduction in TP muscle activation, such that the fascicles actively stretch (eccentric action) and therefore absorb and subsequently dissipate energy. These potentially underlying mechanisms of how energy is absorbed will impact on the mechanics and energetics of locomotion as well as the risk of injury and degenerative damage to both the muscle and its tendon.

The aim of this study was to identify mechanical parameters that correlate to greater energy absorption in the TP MTU between individuals, during early stance. We hypothesised that an increase in energy absorption during foot contact would be the result of greater absorption of energy in the TP tendon, resulting from greater muscle force production (evident by an increase in STJ moments and an increase in TP activity) and maintenance of isometric like behaviour of the TP fascicles. Simultaneous measurements of TP fascicle lengths, measured using ultrasound, were combined with motion capture data of participants walking at a preferred speed on an instrumented treadmill. Musculoskeletal modelling was then used to calculate effective tendinous tissue (series elastic element, SEE) length changes during the STJ energy absorption phase (negative power) in early stance. Intramuscular electromyography of TP was used to determine the onset and total muscle activity generated during the energy absorption phase.

## Results

### Joint mechanics

**Figure 1:**
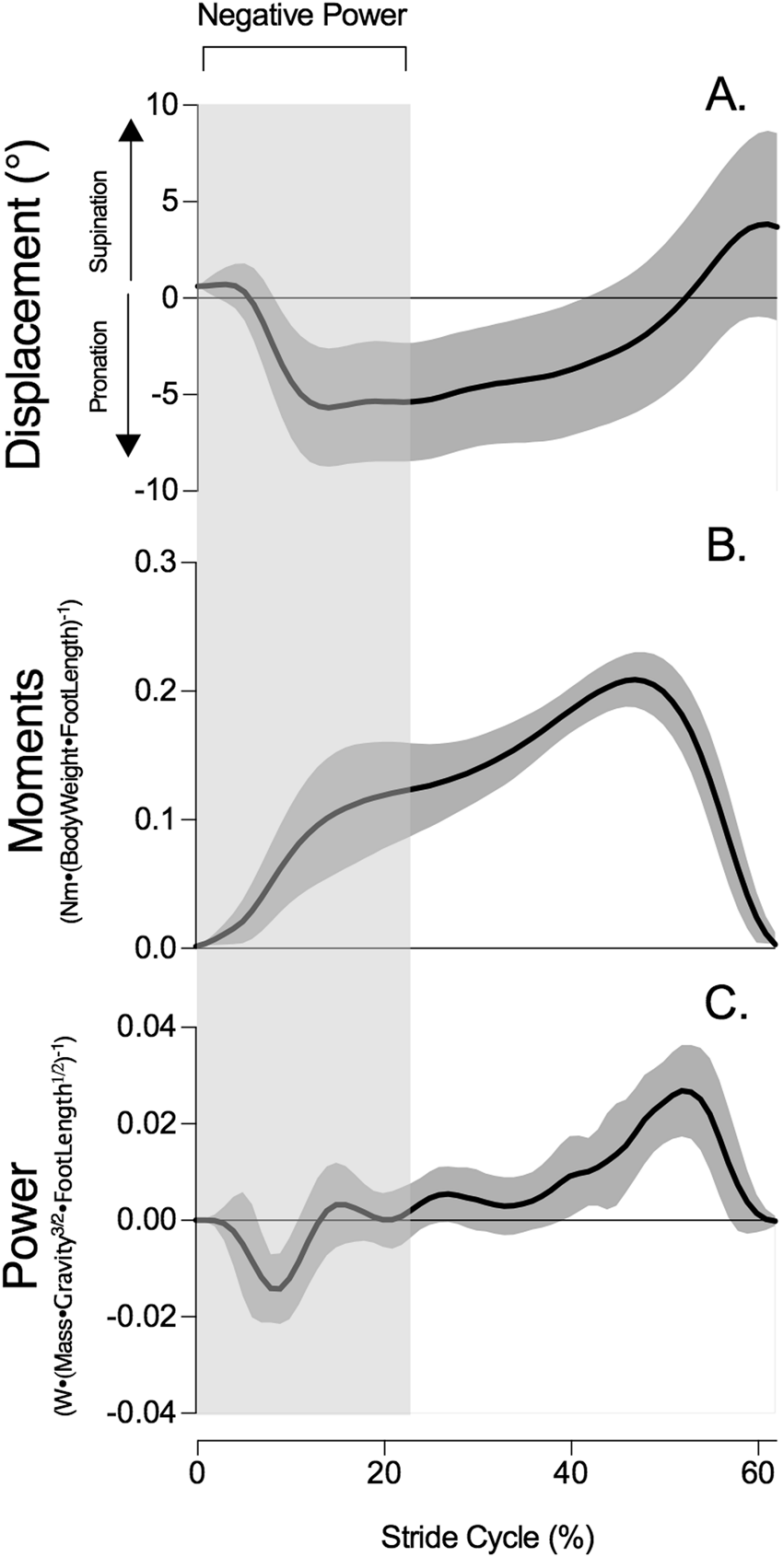
Group mean ± s.d time-series data of subtalar joint mechanics during stance phase of gait at preferred walking velocity including (**A**) rotation displacement (**B**) normalised moments, and (**C**) normalised power. Moments and powers were normalised to body weight (N) and foot length (m). The graphs represent 62% of the stride (corresponding to the average stance time). The shaded area indicates the negative power period.

All participants displayed similar STJ kinematic patterns during early stance. Figure 1 shows that following heel strike, as the STJ pronated, there was a supination moment that resisted pronation and therefore absorbed energy, slowing and ultimately halting the rotation of the foot in a pronated position. The mean duration of a stride and the negative power period were 1.04 ± 0.05 s and 0.16 ± 0.03 s, respectively. The duration of the negative power period was not significantly associated to energy absorption (R^2^ = 0.005), peak supination moment (R^2^ = 0.16), pronation displacement (R^2^ = 0.002) and fascicle (R^2^ = 0.05), MTU (R^2^ = 0.02) or SEE (R^2^ = 0.0001) length change.

### Muscle activation

**Figure 2:**
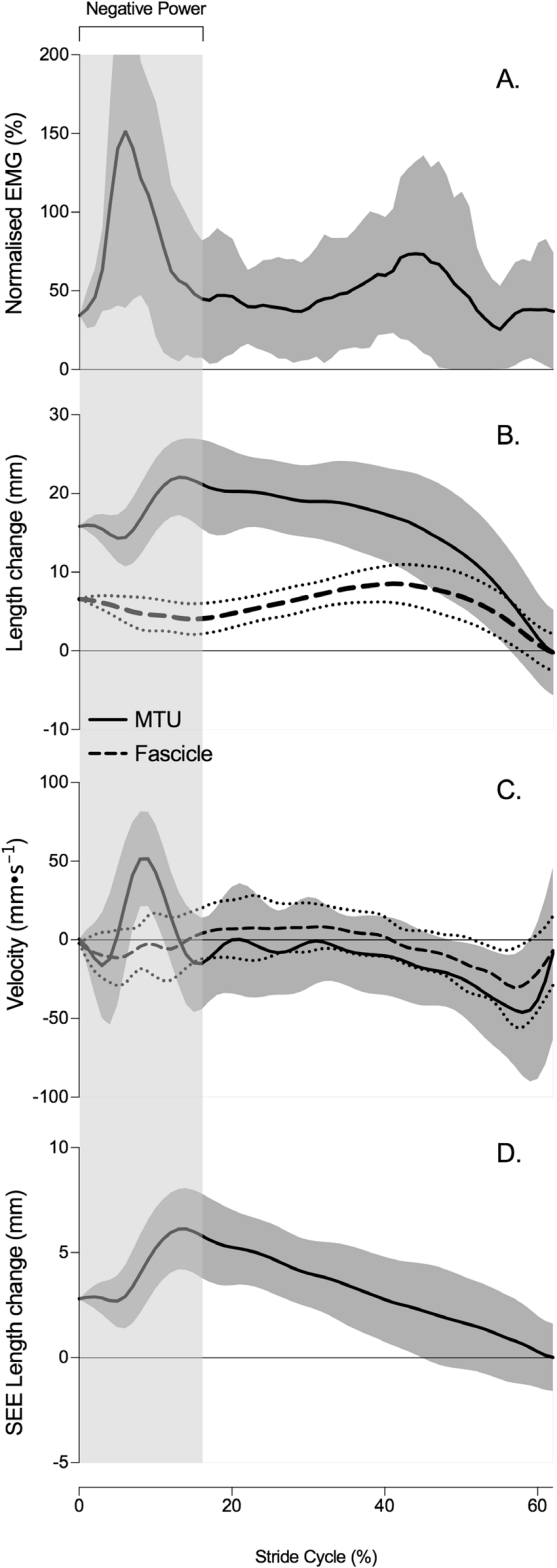
Group mean ± s.d time-series data of tibialis posterior muscle dynamics during stance phase of gait at the preferred walking velocity including (**A**) muscle activity normalised to MVC, (**B**) length change of the muscle-tendon unit (MTU, solid line) and fascicles (dashed) relative to toe off, (**C**) velocity of the MTU (solid line) and fascicle (dashed line), (**D**) length changes of the series elastic element (SEE, tendinous tissue) relative to toe off. The length of zero strain (or zero length) in the MTU and fascicles was approximated from fascicle length at toe off, at the end of stance phase. The graphs represent 62% of the stride (corresponding to the average stance time). The shaded area indicates the negative subtalar joint power period.

During walking, the TP muscle displayed a distinct burst of activity shortly after heel strike at 8 ± 2.7% of the stride cycle, during the negative power phase (Fig. 2a). The EMG area during the negative power phase (absorption activity) was 8.01 ± 2.6%MVC$\cdot$s and was significantly, but only moderately, associated to an increase in peak supination moment (R^2^ = 0.28, P = 0.02). Both muscle activity and onset of activity failed to show significance to any other joint (energy absorption and pronation displacement).

### Muscle mechanics

**Figure 3:**
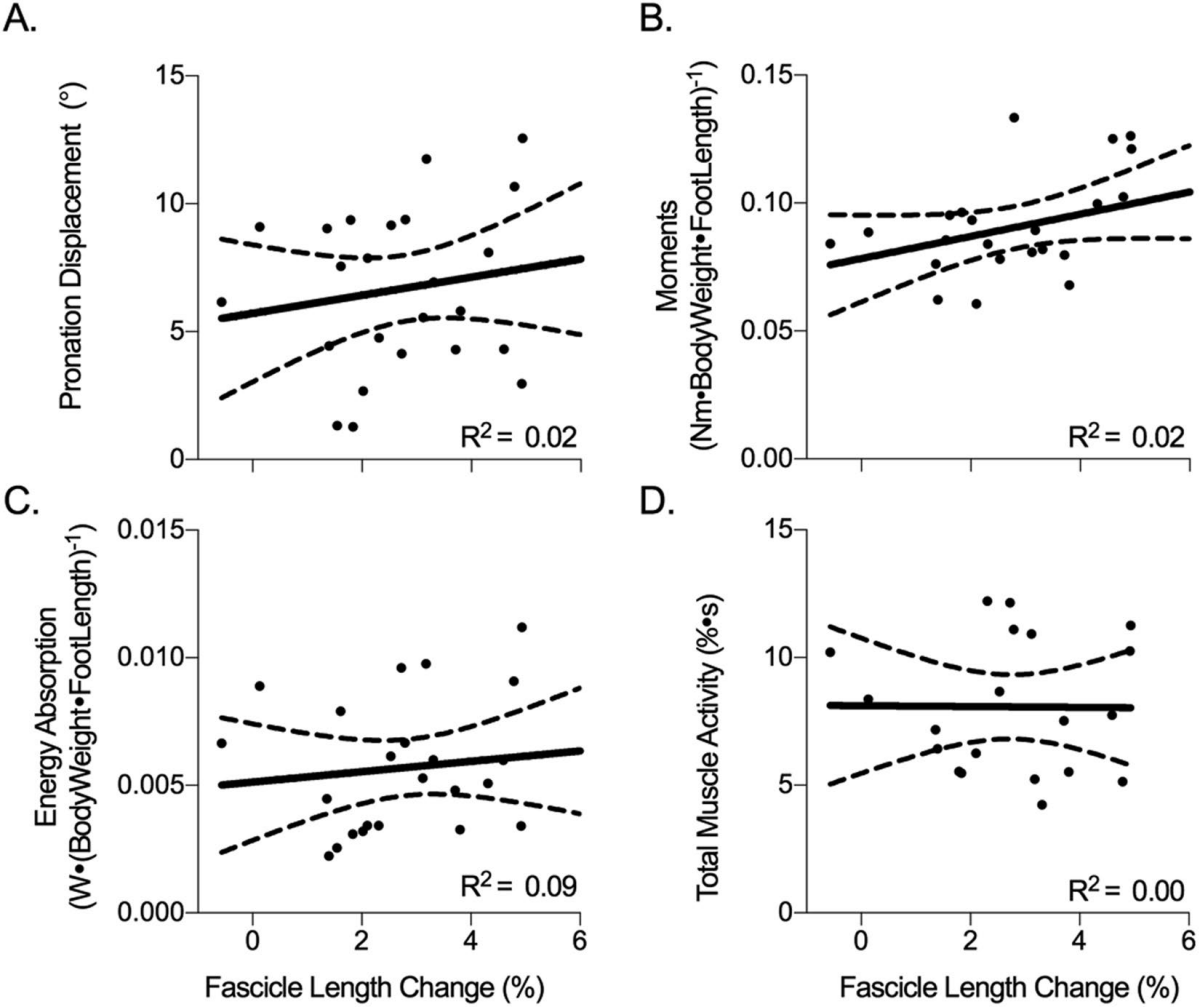
Linear regression analyses of the relationship between tibialis posterior (TP) fascicle length change (%) and (**A**) pronation displacement, (**B**) normalised subtalar joint (STJ) supination moments (Nm∙Nm^−1^), (**C**) normalised (STJ) negative work (J (kg m)^−1^), (**D**) total TP muscle activation during the negative power period. Moments and work were normalised to body weight (N) and foot length (m). 95% confidence interval of the regression lines are shown as dashed lines.

The mean within-examiner coefficient of multiple correlation (CMC) of the TP muscle fascicle length changes was 0.84, suggesting TP muscle fascicle length changes can be reliably tracked during walking^12^. During stance, TP muscle fascicles differed in their pattern of length change to that of the MTU (Fig. 2b). Relative to fascicle length at toe off, the MTU lengthened by 23.2 ± 7.0% as the fascicles remained relatively isometric, experiencing a peak relative shortening of 3.9 ± 2.7% during the negative power phase. Figure 3 illustrates that length change of the fascicles was not significantly associated with any measure of joint mechanics, including energy absorption (R^2^ = 0.09), peak supination moment (R^2^ = 0.02, n = 23), or pronation displacement (R^2^ = 0.02). Length change of the fascicles was not significantly associated to peak plantar flexion ankle moment (R^2^ = 0.004). It therefore seems that fascicle dynamics are relatively invariant across our population and do not contribute to energy absorption at the STJ.

**Figure 4:**
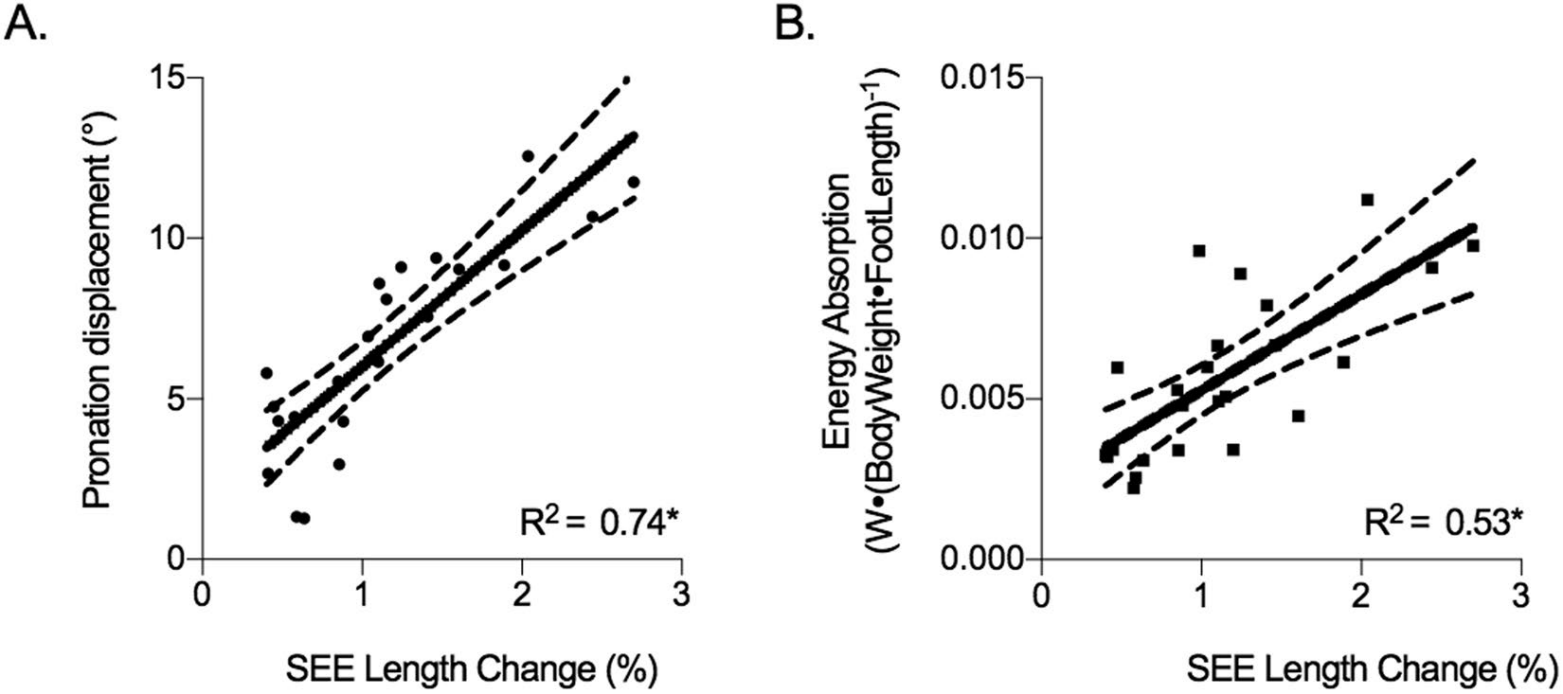
Linear regression analyses of the relationship between tibialis posterior series elastic element (SEE) length change (%) and (**A**) pronation displacement during the negative power period and (**B**) normalised negative work. Work is normalised to body weight (N) and foot length (m). Asterisks (*) represent statistical significance. 95% confidence interval of the regression lines are shown as dashed lines.

Figure 2d shows the estimated length change of the SEE. The SEE initially lengthened 2.1 ± 0.8% relative to SEE length at toe off, after heel strike before undergoing gradual shortening. Length change of the SEE significantly explains 76% of the variation observed in the MTU length change (R^2^ = 0.76, P = < 0.001, n = 24), but demonstrated no association to fascicle length change (R^2^ = 0.02, P = 0.5), indicating that TP fascicle length changes are decoupled to those of the SEE. With respect to joint mechanics, energy absorption and pronation displacement increased as a result of increasing length change of the SEE (Fig. 4). These relationships are considered relatively strong^12^ and significantly different from zero (P = < 0.01). SEE length change explained 53% and 74% of the variance in energy absorption and pronation displacement, respectively (n = 24). An increase in SEE strain was not significantly associated to peak supination moment (R^2^ = 0.03, n = 23) nor plantar flexion ankle moment (R^2^ = 0.004).

## Discussion and Conclusion

The results of this study indicate a relationship between an increase in tendon strain and both energy absorption and total pronation at the STJ. This finding suggests that the TP tendon primarily contributes to the overall energy absorption at the STJ during early stance. Muscle fascicle length changes and surrogate measures of muscle force (ankle and subtalar joint moments together with TP muscle activations) failed to demonstrate any significant relationships with STJ energy absorption or tendon strain, suggesting that increases in energy absorption are not dissipated by muscle fascicle stretch and that variance in muscle force generation may not explain the variance in energy storage between individuals. As such, we deduce that for a normal population, increases in relative TP tendon compliance is responsible for the increased range of pronation and subsequent energy absorption at the STJ in walking. These results are in agreement with recent findings from our laboratory, indicating foot compliance (i.e. foot mobility magnitude) is an essential factor in determining the variance in the energy absorbed at the STJ^13^.

The observed variation in tendon strain suggests that TP tendinous tissue compliance may partly explain why some people pronate more than others during weight acceptance while walking. As such, we envisage that the material properties of the tendinous tissue are likely to vary significantly between individuals. This speculation is supported by the large variations in Young’s modulus (stiffness normalized to tendon cross-sectional area and length) in the TP tendon (0.45 ± 0.16 *GPa*) that has been measured *ex-vivo* ^14^. Other lower limb tendons also show considerable variability in their material properties, including the Achilles tendon (1.167 ± 0.15 *GPa* ^15^) and the tendon of tibialis anterior (1.2 ± 0.15 *GPa* ^16^). Variations may be due to the way in which tendons adapt to long-term and transient mechanical loading, as tendon stiffness can increase with long-term exercise^17,18^ and decrease with long-term disuse and aging^19^. The underlying mechanism is likely to be multi-faceted, however adaptations in dimensions of the tendon (tendon length and cross-sectional area), material properties (changes in the tendon’s underlying microstructure), or both will ultimately result in changes in the elastic function of the tendon.

Tendon compliance can influence the efficiency of movement by modulating the muscle shortening velocity and the tendon’s capacity to store elastic energy. During walking, the pattern of length change of the TP tendon was characterised by an initial period of rapid lengthening corresponding to rapid joint pronation; whilst the fascicles slightly shortened at relatively slower velocities. The length changes of the fascicles were found to be invariant to the joint mechanics, and the consistent pattern of slow shortening velocities whilst the tendon is stretched should enable the muscles to generate forces in an energetically efficiently manner^11,20^. We estimated the TP sarcomere working range during walking to be from 2.4 to 2.6 *μ*m, by dividing our short and long fascicle lengths during stance by an average number of 1.48 × 10^4^ in-series sarcomeres (fibre length [3.78 cm] / sarcomere length [2.56 × 10^−4^ *μ*m]^21^). These values are very close to the optimal length in human sarcomere of 2.7 *μ*m^22^, suggesting that the TP muscle generates force in an energetically efficiently manner. We have previously suggested that the energy stored in the tendon may contribute to powering supination during push-off^3^. The mean estimated tendinous tissue length changes did not show as much shortening during late stance in this study compared to our previously published work, although the contribution is highly variable across individuals. It is likely that greater energy storage in a compliant TP tendon may contribute to greater energy return in late stance, reducing the positive work performed by the muscle and therefore reducing the energetic cost to the muscle. However, as the net work at the STJ joint is close to zero^3^, it is not clear why some individuals would require greater energy storage at contact and then greater power production at push-off.

Strain-induced tendinopathy is common in the tendons of the distal lower limb, particularly in compliant tendons that act as energy recyclers. TP tendon injuries in particular, are related to the mechanical loading of the tendon during walking^23^. *In-vitro* studies suggest that the magnitude of tendon strain is the biggest predictor of time or cycles to failure during cyclic loading, with failure strains occurring between 4 to 12%^24^. This indicates that the strains measured in the TP during preferred walking velocity may, in some people, result in progressive damage to the tendinous material. Furthermore, as the stresses on the tendon (driven by greater STJ moment requirements) increase with walking velocity^3^, the strains imposed on the TP tendon are also likely to increase, potentially resulting in greater potential for micro-damage and degeneration of the tendon. As previously mentioned, tendons can typically adapt to mechanical requirements by modifying their physical structure (e.g. cross-sectional area), therefore tendon strain may be reduced by increasing the stiffness of the tendon (via long-term physical activity) or by reducing the stresses on the tendon^17,25,26^. The requirement for absorbing energy is not well understood, however a recent study^13^ from our group suggests that some modifiable factors such as increasing step width may reduce the moments generated and consequently the requirement for energy absorption at the STJ, which should in turn reduce TP tendon strains. External devices such as foot orthoses have also been shown to alter the activation patterns of the TP muscle^27^ and therefore may also alter the stresses and strains experienced by the tendon.

Although regression models are powerful tools for exploring associations among variables, it must be emphasised that it is not possible to establish a cause and effect relationship using this approach. Variation in the TP tendon compliance was not specifically measured in this study, but postulated from the strains experienced by the tendon that were independent of the forces experienced. Unfortunately measuring the stiffness of the TP tendon *in-vivo* is difficult due to technical issues related to activating the TP muscle and estimating its force generation, which would require directly measuring the STJ joint moment and having a measure of the moment arm of the tendon – both of which are technically challenging. Finally, directly measuring the stretch of tendon as it wraps around the medial malleolus of the tibia would also prove a challenge.

The methodology detailed in this study is well established in previously published literature^3,28^ but is not without certain limitations. During ultrasound recording, it is possible that movement of the ultrasound transducer relative to the muscle of interest, due to bulging of the tibialis anterior muscle and movement of the TP muscle under the tibialis anterior muscle, may cause artefacts to the TP fascicle lengths measured. However, we are confident with our measures based on continuous visibility of fascicles throughout the stride cycle for all subjects. Length changes of the TP MTU are only estimates based on a musculoskeletal model. The MTU length model did not have multiple insertions into the forefoot which makes modelling the precise length changes difficult. However, we are confident that our general findings are sound as the length changes of the MTU were highly correlated to the STJ joint excursion, as should be the case.

## Methods

### Experimental Protocol

Twenty-five participants (13 male, 12 female) with no history of neuromuscular disorders gave written informed consent to participate in the study. The subjects’ average age, height, and body mass were 24.4 years, 1.70 m, 72.5 kg, respectively. The protocol was approved by the University of Queensland Human Research Ethics Committee B and performed in accordance with the Declaration of Helsinki.

Participants were asked to walk barefoot on a force-instrumented tandem treadmill (DBCEEWI, AMTI, USA) whilst kinematic, kinetic, muscle activation and ultrasound data were synchronously recorded from the right leg. For each subject, their preferred walking velocity was selected via a process of incrementally increasing and reducing the speed until the subject consistently identified the same preferred velocity three times. The group average velocity was 1.3 ms^−1^.

### Joint Mechanics

Maharaj *et al*.^13^ have earlier reported procedures for collecting and processing STJ kinematics and kinetics. In brief, angles and moments were calculated using subject-specific models scaled from a custom built multi-segment foot model in OpenSim software^29^. STJ angular displacement was calculated relative to the STJ angle at heel strike. STJ and ankle plantar flexor moment represent the net internal moment produced by the structures at the STJ and ankle respectively. STJ power was calculated as the net moment exerted about the STJ multiplied by the STJ angular velocity. Joint power was integrated with respect to time over the discrete period of negative power using the trapezoidal integration method to calculate negative STJ work. Moments, velocities, power and work were subsequently normalised to body weight and foot length^30,31^ where appropriate. For example, joint moment (Nm) was normalized by dividing the calculated moment by body weight (N) and foot length (m). Foot length has been determined to be the most relevant factor to account for differences in foot dimensions^32^.

### Muscle activation

TP activation was measured using intramuscular electromyography (EMG). Two bipolar fine-wire electrodes (0.051 mm stainless steel, Teflon- coated, Chalgren, USA), each with a 1 mm active recording site, were inserted into the TP muscle using a posterior-medial approach at approximately 50% of the distance between the medial malleolus and the medial joint line of the knee^33^ under ultrasound guidance (Echoblaster, 128, UAB, Telemed, Vilnius, Lithuania). A previously inserted single use 0.25 × 40 mm acupuncture needle (RedCoral, Melbourne, Australia) was used as a visual guide. Verification of the placement of the electrodes in the target muscle was done by ultrasound imaging before the delivery needle was removed. The quality of the EMG signal was assessed by having the participant perform resisted plantar flexion and inversion contractions. Maximum isometric voluntary contractions (MVCs) were collected at the completion of the gait protocol. These were performed with the participants sitting on a bench with their knees extended as they performed supination efforts against the resistance of the tester while being given verbal encouragement. The contractions involved a gradual but continuous 2-s build-up of torque followed by a maximum 3-s effort. Three consecutive maximum efforts were separated by a 1-min recovery period.

Owing to technical difficulties (e.g. signal loss or poor signal to noise ratio), intramuscular EMG data were only fully analysed from 20 of the 25 participants. Raw EMG signals were high pass filtered at 30 Hz and a root mean square (RMS) signal amplitude was calculated using a moving window of 100 ms. Subsequently, the EMG envelope was normalised to the mean RMS amplitude collected from the three MVCs. Muscle activity was calculated over the negative power period for each stride cycle. This measure of ‘absorption muscle activity’ (%MVC∙s) provides insights into the magnitude of activation relative to the time that a muscle is generating force and was calculated by multiplying the mean normalized RMS signal amplitude during negative power period (%MVC) by the mean negative power period duration (s)^34^. By accounting for variations in timing, this measure is an indicator of overall activation cost, which is not reflected using a mean value alone. EMG onset was defined using a threshold crossing which was twice the standard deviation of the EMG above a baseline resting value.

### Muscle mechanics

**Figure 5:**
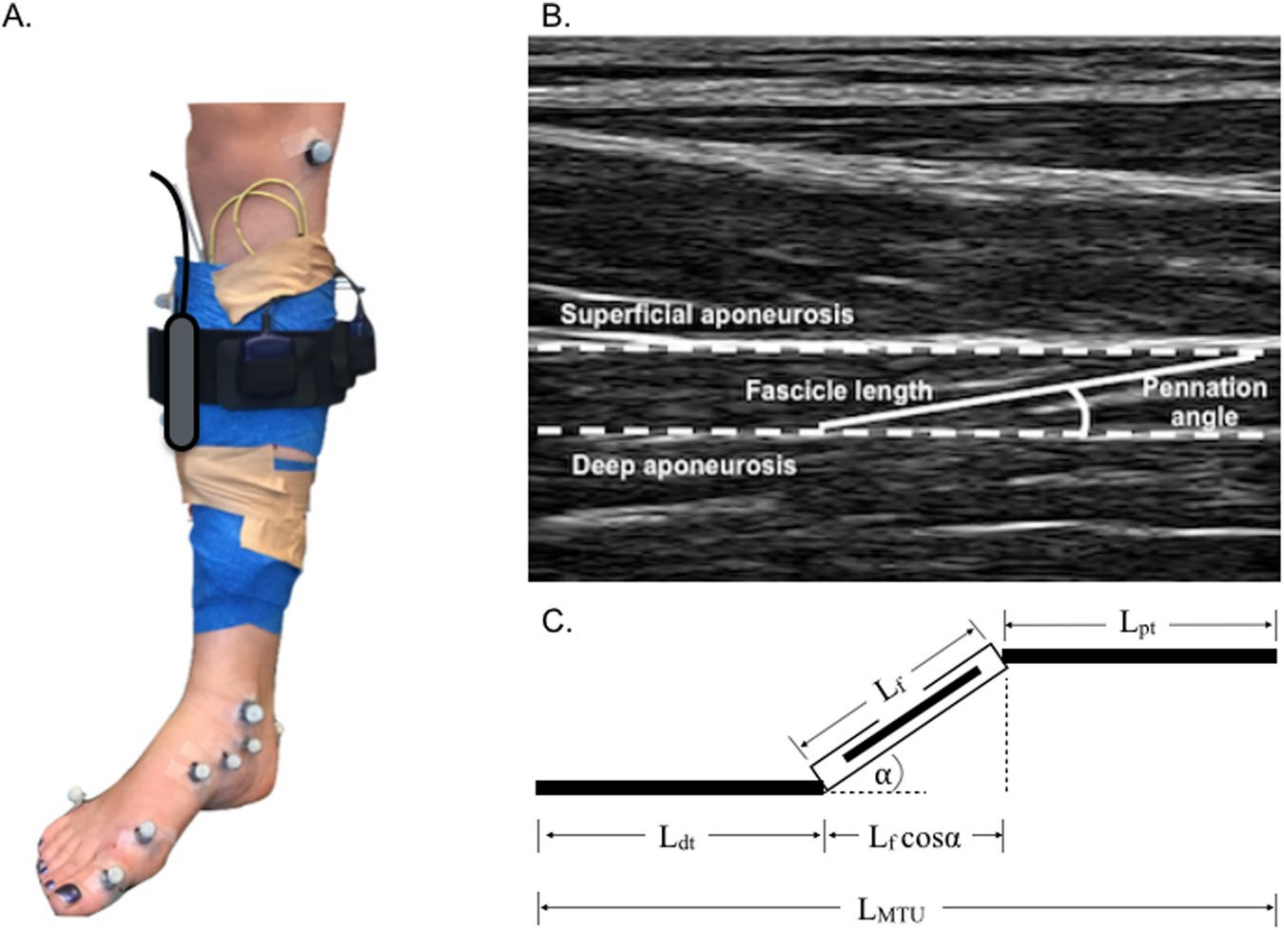
(**A**) Photograph of the experimental setup including location of markers used to track the multiple segments of the foot, fire wire electrodes and the attachment of the ultrasound probe using blue elastic bandage. (**B**) Representative ultrasound image of the tibialis posterior (TP) illustrating the measurement sites for muscle fascicle length (L_f_, solid line), running between the superficial and deep aponeuroses (dashed lines); and pennation angle ($\alpha$), defined as the angle between the muscle fascicle and the deep aponeurosis. (**C**) The musculotendon model used by Fukunaga *et al*.^5^ to estimate series elastic element (SEE) length changes. In this model, the total change in SEE length ($\Delta$ L_dt_ + $\Delta$ L_pt_) is equal to the change in muscle-tendon unit length ($\Delta$ L_MTU_) minus the change in muscle fascicle length in the direction of the SEE ($\Delta$ (L_f_ cos $\alpha$)).

The procedures for collecting and processing ultrasonography data and for calculating MTU and SEE kinematics are reported in Maharaj *et al*.^3^. In brief, muscle fascicle images (Fig. 5a) were collected from the anteriolateral aspect of the leg, lateral to the tibial shaft, which was found to provide the highest quality images of TP fascicles.

MTU and muscle fascicle length change (%) were calculated relative to fascicle length at toe off, which is the time at which minimal forces are likely to be applied to the tendon^6^. Estimates of tendinous tissue elongation were made as previously reported using fascicle length and pennation angle from the ultrasound images, along with the whole MTU length^3^ (see Fig. 5b). SEE length change (%) was calculated relative to the SEE length at toe-off. To test the reliability of the TP muscle fascicle measurements, one stride of each subject was tracked twice by the same assessor. The coefficient of multiple correlation was used to assess the repeatability of the tracking by calculating the overall similarity between the two waveforms^35^.

### Data analysis

Processed kinematic, kinetic, fascicle and EMG data were time-normalised to 101 data points by linear interpolation over a single stride (from right heel strike to ipsilateral heel strike). The negative power phase was defined from heel strike to when STJ power first became positive. Group means were computed from participant means, which were calculated over a minimum of three sequential strides. Outcome variables described as either joint (peak pronation displacement, supination moments, ankle moments, and energy absorption), muscle (MTU, SEE and fascicle length change) and muscle activation measures (EMG onset and absorption muscle activity) were calculated for the negative power period. Least-squares linear regressions were used to determine the effect of muscle activation measures on muscle measures and muscle measures on joint measures. The significance level was set as P ≤ 0.05. Regression models were checked for heteroscedasticity and normality of residuals and any identified outliers were removed. All grouped data are presented as means ± standard deviations.

### Data Availability

The datasets analysed in the current study are available in the UQ Espace repository, http://espace.library.uq.edu.au/view/UQ:673629.
